# To assess whether indoor residual spraying can provide additional protection against clinical malaria over current best practice of long-lasting insecticidal mosquito nets in The Gambia: study protocol for a two-armed cluster-randomised trial

**DOI:** 10.1186/1745-6215-12-147

**Published:** 2011-06-10

**Authors:** Margaret Pinder, Musa Jawara, Lamin BS Jarju, Ballah Kandeh, David Jeffries, Manuel F Lluberas, Jenny Mueller, David Parker, Kalifa Bojang, David J Conway, Steve W Lindsay

**Affiliations:** 1 London School of Hygiene and Tropical Medicine, Keppel Street, London WC1E 7HT, UK; 2 Medical Research Council Laboratories P.O. Box 273, Banjul, The Gambia; 3 National Malaria Control Programme, Banjul, The Gambia; 4 H. D. Hudson Manufacturing Company, 500 N. Michigan Avenue, Chicago, IL 60611, USA

## Abstract

**Background:**

Recently, there has been mounting interest in scaling-up vector control against malaria in Africa. It needs to be determined if indoor residual spraying (IRS with DDT) will provide significant marginal protection against malaria over current best practice of long-lasting insecticidal nets (LLINs) and prompt treatment in a controlled trial, given that DDT is currently the most persistent insecticide for IRS.

**Methods:**

A 2 armed cluster-randomised controlled trial will be conducted to assess whether DDT IRS and LLINs combined provide better protection against clinical malaria in children than LLINs alone in rural Gambia. Each cluster will be a village, or a group of small adjacent villages; all clusters will receive LLINs and half will receive IRS in addition. Study children, aged 6 months to 13 years, will be enrolled from all clusters and followed for clinical malaria using passive case detection to estimate malaria incidence for 2 malaria transmission seasons in 2010 and 2011. This will be the primary endpoint. Exposure to malaria parasites will be assessed using light and exit traps followed by detection of *Anopheles gambiae *species and sporozoite infection. Study children will be surveyed at the end of each transmission season to estimate the prevalence of *Plasmodium falciparum *infection and the prevalence of anaemia.

**Discussion:**

Practical issues concerning intervention implementation, as well as the potential benefits and risks of the study, are discussed.

**Trial Registration:**

ISRCTN01738840 - Spraying And Nets Towards malaria Elimination (SANTE)

## Background

Although progress is being made [1], malaria remains one of the world's greatest childhood killers [2], consumes almost half of the clinical services in Africa (http://www.rbm.who.int), and is a substantial obstacle to social and economic development in the tropics [3,4]. We know from historical accounts that in the 1950s and 1960s malaria was controlled using indoor residual spraying (IRS) with DDT for vector control in many parts of the tropics [5]. DDT was highly effective in reducing malaria infection, but was not used widely in Africa.

Today, Roll Back Malaria is mainly concerned with reducing the burden of disease and is gathering pace. The Global Fund to fight AIDS, Tuberculosis and Malaria (http://www.theglobalfund.org) and the US President's Malaria Initiative (http://www.fightingmalaria.gov) are major players in malaria control and strategies towards elimination in Africa, supported by a myriad of smaller players. The current recommended best practice for malaria control includes the use of Artemisinin-based combination therapies and LLINs. The protective efficacy of LLINs (or insecticide treated nets, ITNs) is well known [6] and they are the cornerstone of malaria control in many African countries, including The Gambia.

Recently, there has been mounting interest in scaling-up IRS, and DDT in particular, for malaria control in Africa as IRS is a passive and a highly equitable intervention [7,8]. In 2009, 32 countries in Africa recommended IRS for malaria control and 13 of those were using DDT (http://www.who.int/malaria/world_malaria_report_2010). In The Gambia, the National Malaria Control Programme (NMCP) implemented DDT IRS over the entire country in 2009. Given this ground swell of support for DDT-based IRS it is surprising there is still a dearth of information about the effectiveness of DDT IRS in Africa; Kouznetsov's statement in 1977 is still valid; [there is a] 'lack of evidence that [IRS] schemes... are contributing to the improvement of population health in highly endemic levels of Africa' [5]. A recent Cochrane review highlighted the paucity of high-quality trials to inform decision making about the use of IRS for malaria control [9], but considered that IRS reduces malaria incidence in areas of stable transmission.

In contrast there is much stronger evidence that ITNs, and by implication LLINs, are protective with a Cochrane Review concluding that in areas of stable malaria they reduce the incidence of uncomplicated malaria in children by 50% compared to no nets [6]. There is a small body of literature suggesting that ITNs may be more protective than IRS in areas of unstable transmission, but conclude that more trials need to be carried out [9]. The only direct comparison made between ITNs and IRS with DDT that we are aware of is in China where both treatments were used separately and had a similar efficacy [10]. Thus no large-scale trials have been carried out to investigate the use of LLINs and IRS together, in comparison with LLINs alone.

Specifically it is key to determine if IRS with DDT will provide marginal protection against malaria over current best practice of LLINs and prompt treatment in a controlled trial, given that DDT is currently the most persistent insecticide for IRS [11]. Since LLINs represent 'current best practice', we propose to measure the marginal impact of IRS with DDT on malaria in communities using LLINs and having access to rapid treatment by conducting a cluster randomised intervention trial in rural villages in Upper River Region (URR), The Gambia, a country where malaria has been declining [12,13]. Here we describe the study design and the methodology used.

### Study objectives

#### Clinical

• Primary objective: To assess whether IRS with DDT plus LLINs provide added protection against clinical malaria in children compared with LLINs alone.

• Secondary objective: To assess whether IRS with DDT plus LLINs provide added protection against anaemia and/or parasite prevalence in children compared with LLINs alone.

#### Entomological

• Primary objective: To assess whether IRS with DDT plus LLINs reduces vector density inside houses when compared with LLIN alone.

## Methods/Design

### Study area and participant eligibility

The study is situated in the far east of The Gambia, in URR, extending approximately 240 to 320 km inland from the mouth of the Gambia River on the Atlantic coast, an area of open Sudan savanna. The climate consists of a single rainy season from May to October followed by a long dry season. This defines the highly seasonal malaria transmission with most malaria episodes experienced during or immediately following the rainy season. This region covers an area of 1995 km^2 ^and is bisected by the river into the north and south banks. The population of the region was 182,586 in 2003, the most recent census, with the majority living in small rural villages in houses made with mud or cement walls and thatched or metal roofs. The study's field station is based at Basse Santa Su town (UTM coordinates 13.3167N, -14.2167W) which is the only sizeable urban area.

Villages or groups of neighbouring villages, will be selected for the study that have > 110 children aged 6 months -13 years and that are ≥2 km from another study village to reduce likelihood of movement of mosquitoes from one cluster to the next. The study cohort of children, aged 6 months - 13 years old in June in Year 1, who are village residents, will be enumerated and an average of 110/cluster will be selected randomly and invited to participate in the clinical surveys and passive case detection (PCD). In order for the results from this study to be as generalizable as possible, no distinctions will be made regarding gender, ethnic group, medical condition or physical health. Children will be included providing their carers/parents give witnessed informed written consent and, in the case of older children, assent is provided. Assurance will also be sought from carers/parents that they expect that the child will remain resident in their village during the transmission season and will not be sent away for example for live stock herding or schooling. Subjects and households are free to withdraw from participating in the study at any time without giving a reason. If consent is not provided then replacement children will be randomly selected from those remaining. In year 2, overage children will leave the study and infants aged 6 to 18 months will be recruited; any other children who have left the study, for whatever reason, will be replaced.

Study subjects will be enrolled in June 2010 in Year 1. Enumeration, recruitment and survey of study subjects and application of the interventions will take place over 2 months, before the start of the transmission season. As is customary in The Gambia, sensitisation will start by discussions with community elders and then the whole study community in order to explain the nature of the study and what will be required during the interventions and investigations. We will seek verbal consent before IRS and verification of positioning of mosquito traps, and written informed consent from the parents or caregivers of all enrolled study children. During these procedures it will be made clear that people will be able to leave the study at any time.

Informed consent will be sought at the village level after sensitization meetings attended by village community leaders and health staff (community level information sheet attached). The key attendees names and roles will be documented for each village. Witnessed verbal consent will be sought from each household head, or representative, before IRS and before population, net and house surveys.

A cohort of 8,000 children will be enrolled from all the clusters to assess the impact of the intervention on malaria. In approximately 50% of clusters, equally divided between the two study arms, 6 rooms will be selected, each with a single male sleeper, to monitor the impact of the intervention on the density of malaria vectors and their infection rate with malaria. These will be randomly selected from households that have consented to join the study, stratified by housing types in the cluster. Amongst the selected households, potential rooms will be randomly selected by numbering, the numbers being placed in a hat and withdrawn blind. If the room owner does not consent or withdraws from the study or moves away, the process will be repeated.

### Design

A cluster-randomized controlled study design will be used, as IRS is a community-level intervention and the village cluster as a suitable unit for randomization. All households in all the clusters will be offered LLIN at no cost to the recipients to enable all residents to benefit from this intervention. The clusters will then be randomized into two equal groups; all households in the villages in one group will be offered IRS and those in the other group will not. It is not possible to conduct the IRS in a blinded manner since a placebo would quickly be identified by the community as non-protective. To assess whether DDT IRS and LLINs combined provide better protection against clinical malaria in children than LLINs alone, 75- 125 children aged from 6 months to 13 years old will be sampled according to cluster size enrolled into a study cohort. These children will be followed during the malaria transmission season in 2010 and 2011. Clinical malaria will be recorded by study staff using PCD in close collaboration with government health workers both at the village and health facility levels. Parasites will be detected by a rapid diagnostic test (RDT, Paracheck Pf, Orchid Biomedical Systems, Goa, India) and treatment will follow government treatment guidelines.

The study cohort will be surveyed for malaria indices (parasite prevalence, anaemia and spleen rate) at the end of both transmission seasons to generate data for the secondary endpoints and also at baseline before the year 1 transmission season. Baseline data will be used to compare the 2 groups before the interventions and end of season survey data will be used to attempt to define a *Plasmodium falciparum *parasitaemia cut-off giving optimum sensitivity and specificity for malaria cases in this setting [14].

**Figure 1:**
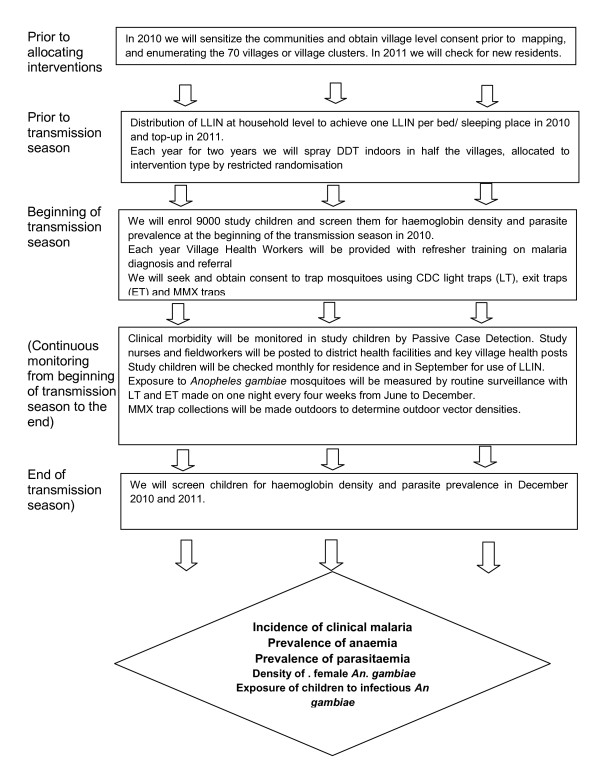
**Schematic of study design**.

Exposure to malaria vector mosquitoes and parasites indoors will be assessed using CDC light traps and window traps every 4 weeks from June to December and every 8 weeks from January to May in at least 6 randomly selected households in 16 village clusters in each arm of the study. This will be followed by detection of *Anopheles gambiae *species, parity status and sporozoite infection. A schematic representation of the trial is shown in Figure 1.

### Randomisation

An equal number of clusters will be randomized to receive IRS, in addition to the LLINs provided to all enrolled households, and in these all households will be treated. In cluster-randomised controlled trials it is particularly important to minimize imbalance for factors known to be highly correlated with the disease outcome; in this case age and access to health facilities and location. At the village level we will stratify by presence of a primary health care centre (PHC) and geographical location by dividing the total area into four with two of equal area on each bank of the river. Stratification for PHCs will also ensure that the larger villages are evenly distributed in the experimental groups. In addition, balanced randomization will be used to ensure that children of similar ages are enrolled at the village level.

Stratified randomisation of villages to the study groups, as outlined above, will reduce the likelihood of chance imbalances between study arms but as only relatively small number of units can be randomized in such a cluster design, both groups cannot be assumed similar for all factors. Baseline data on spleen rates, parasite prevalence and anaemia will be collected on a representative sample of enrolled children in each village at the beginning of the main transmission season in 2010 to assess imbalances in malaria at village level.

Observer bias will be reduced where feasible. Blood films will be read by microscopists blinded to the identity and intervention status of the subjects. Mosquito collector bias will be reduced by using standard light traps and exit traps, which do not rely on the ability of the fieldworker to collect specimens. Trap catches will be examined by a different person to the trap collector and they will be blinded to the trap location. The processes of the interventions, both for LLINs and IRS, will be closely monitored not only for quality but also to document any bias between the villages.

### Interventions

#### a) Indoor-residual spraying

In study clusters randomized to IRS, indoor residual spraying will use a wettable powder of DDT (DDT- WP 75) manufactured to WHO specifications and supplied by Hindustan Insecticide Ltd., New Delhi, India. Indoor surfaces will receive DDT (2 g/m^2^), in May/June, at the start of the main malaria transmission season, in 2010 and 2011. DDT-WP 75, contains 500g of DDT per 670g of product and is packaged in 670g/sachets for making up to 10L with water. The sachets are clearly labelled following WHOPES guidelines (WHO/CDS/WHOPES/2003.7) and are delivered 25/heavy duty carton.

DDT will be stored to maintain efficacy and avoid environmental contamination according to WHOPES guidelines (WHO/CDS/WHOPES/2003.7) and in accordance with the Gambian National Environmental Agency's regulations. Thus the study DDT will be stored in a similar manner to DDT being used currently by the NMCP. The cartons containing individual sachets will be clearly marked with the study name (SANTE) and stored in a locked designated area in the Central Medical Stores until the week before the IRS campaign, each year, when they will be moved to a locked designated area at the Regional Store in Basse. While in the Central and Regional Stores the DDT will be under the control of the NMCP Study Investigators, who will supervise the interventions. The product will be supplied with an expiry date 2-years post-manufacture which will extend beyond June 2011.

During the week before spraying, the sensitization team will inform villages that their village has been selected for IRS, the purpose of spraying, what is required of householders and the excepted IRS date. On the day of IRS, trained spray personnel will inform householders of the spraying schedule and repeat the purpose of spraying and what is required of householders. They will then allow the household time to prepare and vacate the house. Occupants must leave houses before spraying and any rooms occupied by sick people who cannot be moved will not be sprayed. The householder should remove all household items, including water, food, cooking utensils and toys from the house. When possible furniture should be moved outside; if this is not possible, it should be moved to the middle of the room to allow easy access for spraying walls and covered. All wall hangings should be removed. When water-jars cannot be removed they should be covered. Pets and domestic animals should be tethered away from the house. Rooms will not be sprayed if people or animals are present, if household items are not correctly removed or positioned or if the walls are covered with gloss paint. Thatch ceilings also will be sprayed but not tin or hardboard ones. Data will be collected on interior wall surfaces at baseline. Householders will be allowed back into their houses after spraying and will be asked to mop any excess solution from the floors and wash the floors with water before they allow children back into the house. They are requested not to wash, paint or re-plaster sprayed walls at least until the end of the malaria transmission season.

#### b) Long-lasting insecticidal mosquito nets

LLINs distributed during this project will be Olyset Nets, Sumitomo Chemicals. These meet WHO specifications (http://www.who.int/whopes/Long_lasting_insecticidal_nets_Aug09.pdf) with permethrin at 2% w/w incorporated into polyethylene fibres giving adequate release of permethrin for up to five years. Recipients will be offered a choice between the "large" conical (1250 cm circumference, 65 cm top, 250 cm high) and the "family" rectangular (160 cm wide × 180 cm l × 150 cm h) models. Olyset nets are supplied in individual sachets labelled by the manufacturers and they will be distributed to recipients in pre-opened individual sachets in May 2010, at the beginning of the main transmission season.

Olyset LLIN will be stored at ambient temperature in locked rooms in Banjul and Basse and will be under the control of the NMCP Study Investigators. LLIN distribution will conducted in collaboration with the Regional Government bednet distributors at no cost to the recipients. In advance of distribution, the number of beds/sleeping places and the number with LLIN in excellent condition will be counted. Distribution will be at the household level to the household head or representative to achieve one LLIN per bed/sleeping place. Net use by study children will be monitored during the middle of the transmission season in 2010 and 2011. Government Roll Back Malaria information, education and communication procedures will be followed to encourage correct net use and maintenance.

### Clinical data collection and patient treatments

Passive surveillance for malaria will be maintained throughout both transmission seasons. Parents/guardians will be encouraged to take their child to the health centre or health post identified as being closest to their home at any time their child becomes unwell. The Village Health Workers (VHW) who run the health posts will use RDTs in their diagnosis of malaria and treat malaria in children who are enrolled into the study. Nurses placed in strategic health facilities will also perform a RDT in children who present directly to them who are enrolled into the study. Project field workers or nurses will visit each VHW regularly, at least once a week, to record children with malaria episodes.

The parents of any child treated for malaria at a clinical survey or during the passive surveillance will be asked to bring their child to the nearest health facility if the child does not show improvement within 48 h. When such study subjects report to a health facility government nurses will treat the child following Gambian government guidelines and inform the study nurse stationed in that facility. The study nurse will inform the study physician of the case within a minimum of delay. If such study subjects report to the VHW, he/she will advise them to report to the nearest health facility. Referred study subjects will be compensated for transport to the health facilities.

The end of transmission season survey in December 2011 will be the final study visit for all enrolled children and also the final study visit to the villages. The final survey will be similar to the baseline and end of the first transmission season survey in the previous year.

During the course of the study participants are free to receive medication from health personnel outside the study team. During the clinical surveys, iron supplementation will be given to any enrolled child with anaemia (Hb < 9 g/dL). Artemether-lumefantrine (Co-Artem) will be given to any child with a temperature of ≥37.5°C or a history of fever in the past 48 h, and *P. falciparum *parasites detected by RDT in the absence of other detectable cause of fever. The parent/carer of any child we treat for malaria will be asked to bring their child to their nearest health post or facility if the child does not make a recovery within 48 hours. Children with severe malaria will be referred to Basse Health Centre for treatment.

During the passive surveillance for malaria, parents/carers of study children will be encourage to take their child to the nearest VHW or Health Facility if the child is unwell. In both cases children with symptoms suggestive of malaria will be tested for the presence of parasites using RDTs. Treatment for other conditions will be carried out in accordance with national guidelines and/or referral to Government health facilities.

### Entomological collections

Exposure to mosquitoes will be measured by surveillance with light traps and house exit rates estimated using window traps from June to November each year. To obtain the primary entomological endpoint light traps will be used. They are routinely used in The Gambia and are unaffected by the presence of treated nets [15]. A CDC miniature light trap will be positioned close to one sleeper protected with a LLIN. Two estimates of EIR and the number of sporozoite infective bites/child/season will be calculated: one based on light traps collections and one from exit traps. Parity will also be assessed in female mosquitoes.

### Clinical evaluations

The main morbidity outcome will be incidence of clinical disease assessed by PCD; data from this will provide the primary endpoint of the study. PCD is considered to generate information of more direct relevance to public health than active case detection and is thus more suitable for evaluating population level malaria interventions [16]. Mild malaria and parasitaemia is common in all children in this area of highly seasonal malaria [17,18] and we will enrol children aged 6 months - 13 years. We previously developed a system in Gambian villages where a trained MRC nurse provided diagnostic support to the established VHW in the study villages [19]. Following this experience, in this study the nurses will provide training and ongoing support to the VHW for diagnosis of malaria in consulting study children who report with a history of fever in the last two days using RDT and treatment of malaria. Study nurses at the health facilities will document episodes of malaria in study children who present directly to them on the Case Report Form (CRF). At the health facilities malaria diagnosis will also rely on the use of RDT but, in addition, thick blood films will be made for later staining with Giemsa and microscopy to estimate parasite density in this subset of cases. To facilitate documentation of all clinical events (i.e. clinical consultations by study children), all enrolled children will be issued with enumerated photo identity cards; the study number and village will be recorded for all clinical events on the CRF. The project field workers or nurses that visit each VHW will complete the CRF for all study subjects that consult with the VHW.

All children present in their villages at the surveys will be clinically examined for obvious symptoms and signs of illness, temperature and spleen enlargement. A sample of all the study children, at least 50/village cluster will be randomly selected stratified by age, and these as well as those reporting fever in the last 48 hours and/or with a temperature of ≥37.5°C will be finger pricked for immediate measurement of anaemia and presence of parasites by RDT. Anaemia will be assessed by measuring haemoglobin concentration using a spectrophotometer (HaemoCue^®^) in the field.

The primary endpoint selected is the definition of malaria used in The Gambia in research studies and in the National Treatment Guidelines [18,20,21]. However, a parasite cut-off level may increase specificity but the gain in specificity cannot be known in advance with the changing infection rates [12,13]. We thus also intend to use the logistic regression method of Smith and colleagues [22] to examine the effect of parasite cut-off on the specificity and sensitivity on the definition of clinical malaria. This method has recently been endorsed by a WHO study group for use in studies defining malaria vaccine efficacy [14].

### Entomological evaluations

Exposure to mosquitoes will be measured by surveillance with light traps and house exit rates estimated using window traps to obtain the primary entomological endpoint [23]. A CDC miniature light trap will be positioned close to one sleeper protected with a LLIN. Two estimates of EIR and the number of sporozoite infective bites/child/season will be calculated: 1 based on light traps collections and 1 from window traps. Parity will be recorded separately from both trap types.

### Study procedures and evaluations

In advance of the spray campaign in year 1, spray operatives and supervisors will be trained on the WHO methods for IRS with DDT for two weeks by the NMCP vector control officer seconded to the project and an internationally recognized IRS consultant. They will be equipped with personnel protective clothing, including boots, long-sleeved cotton overalls, hat, gloves, mask and boots and trained in the maintenance and use of these. Spray operatives will use spray pressurized canisters made to WHO specifications WHO/VBC/89.970 (Hudson X-Pert^® ^Sprayer, 15L model 67422AD with stainless steel nozzle (8002E), flat fan pattern, 80° angle, flow rate 0.76L/min at 45psi pressure). They will be rigorously trained in the use of these and their maintenance. A 2-day refresher training will be provided before the spray campaign in year 2. Any remnant in the sprayers at the end of daily spraying will be collected following the "Progressive Rinse" method and recycled to minimize waste (http://www.hdhudson.com/global-public-health/newsletters/technical-information accessed 9th June 2011).

It is intended that both IRS and LLIN distribution will be completed in May both years. In year 2 all households in the arm randomized to this intervention will receive IRS as in year 1. LLIN distribution in year 2 will be to residents who have relocated to study villages since year 1.

Village health workers will be provided with refresher training on diagnosis of malaria, the use of RDT and study procedures in July 2010 by the study physician in collaboration with study nurses and the community health nurses. The methods used will be informed by ongoing studies in The Gambia on the introduction of RDT at all levels of health service. Refresher training will be provided in 2011 before the transmission season.

Movement of children will be assessed at monthly intervals on brief questionnaires to estimate the time at risk and bednet usage will be assessed during these house visits in August each year.

Thick blood films will be stained with Giemsa and examined under 1000-fold magnification by trained, experienced microscopists. Parasite counts will be recorded per high power field and 100 fields will be counted before a slide is declared negative. Parasite density will be estimated assuming that one parasite per high power field equals 500 parasites/μl [24]. Two slides will be prepared from each subject and read separately by two experienced microscopists; discrepancies will be resolved by a third reader.

Mosquitoes will be identified by microscopy and the numbers of *An gambiae *s.l. and other species recorded. The presence of sporozoites in *An gambiae s.l. *will be identified using an enzyme-linked immunosorbent assay [25] and *An. gambiae s.l. *females will be typed to the species by PCR [26].

### Safety considerations

There are no apparent risks to the safety of individuals or communities in this study. Permethrin-treated long lasting nets and DDT IRS has been fully evaluated by the WHO Pesticide Evaluation Scheme and approved for vector control (http://www.who.int/whopes/en/), and the products will be used in compliance with their recommended use and guidelines.

All children enrolled in the trial will have access to malaria diagnosis and treatment according to Gambian National Treatment Guidelines and all visits to the VHW or health facilities will be documented on the CRF.

In the case that a participant develops a serious adverse event (SAE) during the course of the study this will be captured on the CRF for PCD if it is associated with a confirmed or suspected malaria episode. When a study child consults with the VHW with a severe illness that is not malaria, this will be captured in the VHW daily log. The CRF for PCD or the details from the VHW daily log will be submitted to the study physician by the nurse or fieldworker responsible for the village-cluster on a weekly basis. If it is a serious adverse event (SAE) the study physician will record and manage the SAE in accordance with Good Clinical Practice (GCP).

The study physician will check all CRF regularly, at least once a week, and will inform the IDMC of the numbers of malaria attacks and SAE by village as required by the ID. Suspicion of potential harm to participants or the environment caused by the interventions may lead to discontinuation of the study. Excessive clustering of SAE by village(s) will be reported to the study's Data Monitoring and Ethics Committee.

If an individual wants to terminate his/her participation, no further follow-up will be performed. In the case of a study child leaving during year 1, they will be replaced in year 2 of the study (June 2011). There will be no replacement during the surveillance period either year. If a household withdraws consent, no further follow-up will be made and there will be no replacement. If a village opts out of the study before July in either year replacement by a neighbouring village will be considered.

### Study endpoints

#### a) Clinical

Primary: Incidence of clinical episodes of malaria presenting at health facilities defined as a child with an axillary temperature of ≥37.5°C or a history of fever in the past 48 h, together with the presence of *P. falciparum *parasites of any density detected by microscopy and/or RDT in the absence of other detectable cause of fever.

Secondary: (i) mean haemoglobin concentration in children in the two study arms measured in the end of the transmission season survey, (ii) parasite prevalence in children in the two study arms measured at the end of the transmission season.

In addition we will also calculate the incidence of malaria defined by history of fever or temperature of ≥37.5°C and a *P. falciparum *parasitaemia cut-off to give optimum sensitivity and specificity in this setting, and number of children with enlarged spleens at the end of the transmission season.

#### b) Entomological

Transmission parameters in the two arms of the study will be estimated from measurements made throughout the transmission season.

Primary: The mean number of female *An. gambiae *s.l./light trap/night inside sleeping rooms.

Secondary: The entomological inoculation rate (EIR) in each study group will be estimated as the mean number of sporozoite infective bites/child/season)

Tertiary: (i) The proportion of mosquitoes exiting houses (since DDT has repellent properties), (ii) the relative abundance of different members of the *An. gambiae *complex in light trap & exit traps collected in each arm of the trial, (iii) the relative abundance of *An. gambiae s.l. *collected outdoors (iv) the parity of *An. gambiae*, to measure mean population longevity.

### Sample Size Rationale

#### a) Clinical

**Figure 2:**
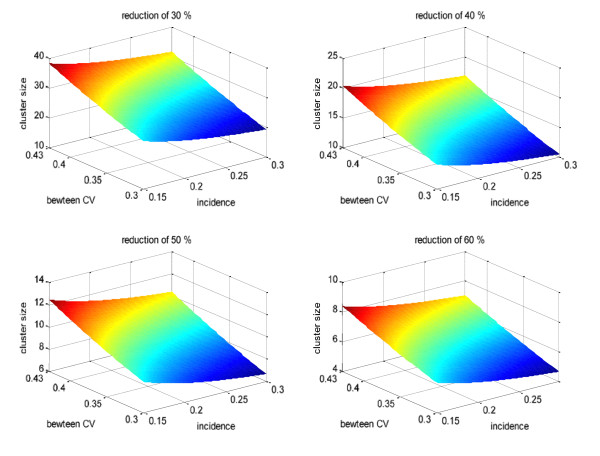
**Sample size estimations at varying effect, incidence and coefficient of variation (CV)**.

Both LLINs and IRS alone reduce malaria morbidity by approximately 50% [5,6]. The combined impact of these two interventions is unknown. If the effects were simply additive a mean reduction of 75% could be anticipated for the combination, but the effects may be synergistic (e.g. by having a mass killing effect) or antagonistic (e.g. the repellency of DDT reducing *Anopheline *house entry and contact with LLINs). To cover these possibilities we expect that LLINs will reduce incidence by 50%, with LLINs and IRS combined reducing the residual incidence by 30-60% (i.e. 50% vs 65-80% fewer clinical cases of malaria). A study of 3-60 month old children during the end of the rainy season (Sept-Dec) in the URR in 2007 found a seasonal incidence of 0.79 (95% CI: 0.58-1.08) cases/child-years by PCD (Bojang pers. comm.); ITN prevalence was 50%. We estimate conservatively that increasing LLIN provision to > 80% and inclusion of older children will reduce incidence rates by about 60-80%, so incidence rates of 0.30 (LLINs) to 0.15 cases/child-years (LLINs & IRS) have been included in the sample size estimations. The study will also record PCD over two high transmission seasons, not 1 as in the Bojang study, further enhancing our ability to detect differences between study arms. Estimations for k, the corrected coefficient of variation to adjust for between cluster variation (based on within and between cluster variation) [27], from a cluster trial conducted in the Central River Region of The Gambia in 2006/7 (K. Bojang personal communication) varied from 0.31-0.43 and has been used to explore sample sizes (Figure 2). Two of the 5 districts in URR have 70 villages suitable for the trial with over 100 children aged 6 months-14 years (predicted from the 2003 National Census) and could accommodate an average cluster size of 110 children (range 75-125). We plan to follow these children for 22 weeks covering the peak malaria incidence season each year, which, allowing for a 20% drop out rate over the 2 years, yields 74.4 child-years per cluster. The graphs below indicate a sample size of 5-39 clusters per arm (Figure 2). Since estimates for incidence and drop-out rate are conservative and the value of k may also be reduced by the stratified design, we will recruit 35 clusters in each arm of the trial.

Data from the same cluster trial quoted above was used to explore sample size considerations for the parasitological and anaemia, endpoints. Considering parasitaemia as a proportion and haemoglobin as a concentration with 35 clusters of 110 children each suggested that sampling half the child cohort would have 80% power to detect a 30% reduction in parasitaemia and a 0.5 mg/mL reduction in haemoglobin at 95% confidence.

#### b) Entomological

Previous studies with experimental huts indicated that DDT IRS deterred 60-70% of mosquitoes from entering houses [28]. LLINs were not associated with a deterrent effect in Gambian houses [15]. Based on a study using light traps in an adjacent study area in 2005, we expect that the mean number of female *An. gambiae s.l. *per trap will be 4.8 (with a coefficient of variation, k = 0.44). In order to demonstrate a 60% reduction in hungry indoor mosquitoes (i.e. unfed *An. gambiae*) associated with DDT IRS, with 90% power and at the 5% level of significance, we would require 8 houses in each cluster and 12 clusters in each arm of the trial over 2 years [27]. Since the number of mosquitoes rise and fall during the rainy season we propose to sample from each house 6 times each year. To allow for loss to follow-up due to people moving house during the study period we propose to include at least 8 houses in each cluster and 15 clusters in each study arm for 2 years.

### Data handling and record keeping

The demographic data will be recorded by fieldworkers and the clinical data by study nurses on standardised data forms. Each participant will have a unique identification (ID) number. All data recorded on individuals will be made by recording the anonymous ID.

Data forms will be scanned to produce electronic copies and into the data set using Snap Survey (Version 10, Snap Survey Ltd., Haymarket, London, UK) software and verified against electronic copies of the original forms by data entry clerks to produce 2 independently verified versions. These will be combined and errors corrected to produce a single dataset. This will be submitted to consistency checking by generic and study specific algorithms designed to identify sources of error. When inconsistencies are found, these will be checked against the original forms and subsequently amended in the dataset. Errors will be corrected when possible, with checking in the field when necessary and possible, to produce final datasets.

All forms with subject names and/or clinical data will be kept in a locked cabinet, when not in use, and the key kept by the study coordinator or delegate. These datasets will be, password protected. Data will be stored for at least 10 years. The clinical data will be kept separately from that containing personal information. All forms and datasets, apart from those for enumeration, will identify subjects by their subject specific ID and names will not be collected or entered. After the collection and verification in Snap Survey data will be moved to MRC's SQL server 2008 SWIFT. These datasets will be, password protected and only accessed by senior study staff and the study Data Manager. Data will be stored for at least 10 years.

The Principal Investigator (PI) will maintain appropriate medical and research records for this study in compliance with GCP and regulatory and institutional requirements. Authorised representatives of the sponsor, the ethics committee(s) or regulatory bodies may inspect all documents and records required to be maintained by the investigator. The PI or designee will ensure the access to facilities and the records.

The PI or designee will document and explain any deviation from the approved protocol on the CRF, where appropriate, and record and explain any deviation in a file note that will be maintained as an essential document. Deviations from the protocol, GCP or study specific requirements that might have an impact on the conduct of the study or the safety of participants will be reported within 15 working days to the sponsor and relevant Ethics Committee, as appropriate. Any other protocol deviation will be reported to the local EC together with the annual report.

### Analytical plan

#### Outcome 1 - malaria morbidity

The primary endpoint is a comparison of the incidence of clinical episodes of malaria in children in the 2 intervention groups, measured by PCD. After a case of malaria is treated, time of observation will be censored for 3 weeks and further attacks of malaria during this period are not considered. History of travel away from the village for prolonged periods will be captured by monthly surveys and time at risk will be censored for such periods. In addition, malaria cases in children who resided outside their study village for more than half the elapsed study period at the time of illness will be censored. An initial unadjusted analysis will be based on comparisons of the incidence rates between the 2 groups. Incidence rates will be calculated for each cluster and the ratio of their un-weighted means will give an estimate of the intervention effects. Confidence intervals for the intervention effect will be obtained using the approximations given by Bennett [29] and tested using a bootstrapped [30] confidence interval. The incidence rates for each cluster will also be used to perform a hypothesis test based analysis between the 2 groups using a 2 sample unpaired t-test. It is likely that the rates will be highly skewed and if necessary the rates will be transformed. Using the incidence rates as a response, linear regression can be used to adjust for cluster level covariate effects. If the assumptions of normality and constant variance cannot be met, a non-parametric test (Mann-Whitney) will be used to compare the 2 groups. The cluster randomised trial will result in a multi-level model with individuals within village. Individual level covariates will be modelled using random effects Poisson regression which will be fitted using GLLAMM, a Stata program to fit generalized linear latent and mixed models GLLAMM [31], to allow for the clustering effect of village, compound and the effect of repeated episodes in the same child. Year will be included as a fixed effect which will allow any interaction with the intervention to be quantified. Other potential confounders such as house architecture, bednet use and recent antimalarial treatment will also be adjusted for in the analysis. Should the data, be zero-inflated or under/over dispersed modifications to the underlying distributional assumptions will be examined. The time to first infection will be examined using a survival analysis approach. Initial unadjusted analysis will use Kaplan Meier curves to compare the probability of subjects in the two arms becoming infected as the malaria season progresses. A Cox regression frailty model will be used to adjust for covariates, allowing for the clustering. The location of the infected subjects will be known to the household level and this information will be used to examine the spatial distribution of infection over time. Within the larger villages formal methods such as k-means fuzzy clustering [32] will be used to quantify clusters of infection.

#### Outcome 2 - malaria transmission

Differences in malaria transmission experienced in the 2 groups will be made by comparing the mean number of mosquitoes caught indoors in houses between the intervention groups. Generalised estimating equations will be used to estimate differences in numbers of indoor-resting mosquitoes, adjusting for repeated measures within clusters and possible covariates.

#### Secondary endpoints

For the secondary clinical endpoints we will compare: (i) mean haemoglobin concentration in children at the end of the transmission season, (ii) incidence of malaria defined by history of fever or temperature of ≥37.5°C and a *P. falciparum *parasitaemia cut-off defined to give optimum sensitivity and specificity in this setting, (iii) parasite prevalence in children at the end of the transmission season, (iv) number of children with enlarged spleens at the end of the transmission season. In general, all quantitative outcomes will be compared using appropriate summary statistics, such as mean differences or risk ratios. Hypothesis testing will be based on a two sample unpaired t-test, adjusting for possible co-variates. If the data do not satisfy normality and constant variance assumptions, an appropriate non-parametric test will be used. Both linear regression (for continuous variables such as haemoglobin levels) and logistic regression (for dichotomous variables such as presence or absence of parasitaemia) will be used to quantify the group differences allowing for individual level covariates (such as age, house design, bed net use) using random effects to allow for clustering.

### Timetable of activities

The timetable of activities is shown in Table 1.

**Table 1:** Timetable of activities

Activity	J	F	M	A	M	J	J	A	S	O	N	D
2010												

Senior staff recruitment	X											

Ethical approval	X	X										

Order and shipment of equipment		X	X	X								

Field staff recruitment & training			X	X								

Training of sprayteams, planning IRS				X								

Sensitisation of study population			X	X	X							

Enumeration & house selection for entomology						X						

House spraying & LLIN distribution					X	X						

Selection subjects, PCD, & travel monitoring						X	X	X	X	X	X	

Entomological surveys						X	X	X	X	X	X	

Clinical & bednet surveys						X					X	X

Data programming and entry					X	X	X	X	X	X	X	X

2011												

Steering Group Meeting in The Gambia			X									

ELISA and PCR assays	X	X	X									

Data entry cleaning & report writing	X	X	X	X								

Sensitisation of study population			X	X								

House selection for entomology				X	X							

House spraying & LLIN distribution					X	X						

Replace subjects, PCD & travel monitoring						X	X	X	X	X	X	

Entomological surveys						X	X	X	X	X	X	

Cost data collection									X	X		

Clinical & bednet surveys											X	X

Data analysis											X	X

Data entry						X	X	X	X	X	X	X

2012												

Data entry, cleaning & analysis	X	X	X	X								

Steering Group Meeting in the UK				X								

Report findings to study communities					X							

Data analysis & report writing					X	X	X	X	X			

Cost effectiveness analysis					X							

Final report to the Steering Committee										X		

Report findings to Gambian Government											X	

Scientific paper writing			X	X	X	X	X	X	X	X	X	

### Ethical approval

This study is conducted in accordance with the principles set forth in the ICH Harmonised Tripartite Guideline for GCP and the Declaration of Helsinki in its current version, whichever affords the greater protection to the participants. It was approved by the Gambian Government/MRC Laboratories Joint Ethics Committee first approved on the 12th August 2008 (ref: L2009.15, L2010.19; SCC1128) and the London School of Hygiene and Tropical Medicine Ethics Committee approved on the 16th September 2009 (ref: 5592).

## Discussion

As enthusiasm for malaria control in Africa gathers pace, there is an urgent need to evaluate the effectiveness of multiple interventions. This study evaluates the efficacy of the 2 most common vector control tools used for malaria control: IRS and LLINs. This study sets out to examine whether there is an additional benefit of using IRS in combination with LLINs compared to current best practise, the use of LLINs alone. It is designed to measure whether the double intervention will provide greater protection against malaria incidence by substantially reducing transmission.

The main morbidity outcome will be incidence of clinical disease assessed using PCD. PCD is considered to generate information of more direct relevance to public health than active case detection and is thus more suitable for evaluating population level malaria interventions [16]. It is especially relevant as many African countries, the Gambia included, have launched policies that malaria should be eliminated as a public health problem. All the clinical outcomes will be measured in a cohort of children aged 6 months to 13 years of age following the age pattern of disease and infection in this area. In areas of high malaria transmission the main burden of paediatric malaria is borne by children under 5 years old, but this pattern shifts to include older children and adolescents in areas with lower levels of transmission for example Ndiop in Senegal and the Farafenni area of The Gambia [17,33]. This trend is increasing as malaria decreases for example a recent study in the Gambia and Guinea Bissau, which included a site in the URR, found parasite prevalence highest in the 11 - 15 years olds [34]. The lower limit of 6 months has been selected in the current study for cultural and clinical reasons and the upper limit corresponds to the age when most children move to middle schools (Upper Basic) which are often further away from their village. In addition to quantifying malaria incidence and parasite prevalence we will measure anaemia in both the community surveys and at the time of clinical attacks and all subjects with Hb < 8 gm/dL will receive treatment. In addition to the clinical benefit, malaria is a major risk for anaemia in these populations [35], as other causes, such as hookworm infection are infrequent, and documented changes in anaemia will complement the main outcomes.

There are no apparent risks to the safety of individuals or communities in the use of these interventions. Permethrin-treated long lasting nets and DDT-WP have been fully evaluated by the WHO Pesticide Evaluation Scheme and approved for vector control [11]. The biggest hazard associated with the trial is the leakage of insecticide, particularly DDT, into the external environment. National guidelines for insecticide transport, storage and all operational procedures will be strictly followed to ensure that this remains unlikely. However, in The Gambia about 5% of rural traditional houses are destroyed each year during the rains [36], suggesting that some DDT may become disseminated into the environment.

LLIN are similarly beneficial at the individual level, and at the community level where high coverage is attained. However, in The Gambia no community protection has been found with ITNs [37-39] as it has in other parts of Africa [40,41]. Study children will benefit from a health check at the surveys and at both the village and health facility levels clinical services will be supported by training and the presence of study nurses in addition to government staff.

It is also important that the correct normal practice of parents/carers in the case of their child becoming sick is not altered in a detrimental fashion by their child being enrolled in the study. For example a parent/carer may delay taking a child to the nearest health facility/post if they think that a study nurse is visiting their village the next day. Both the provision of fares when a children reports sick at a health facility and emphasis of the vital role of parent/carers during enrolment and the conduct of the study will help to mitigate against this.

There is currently a rush for DDT IRS in many African countries, yet we lack the evidence of the protective efficacy of this intervention against clinical malaria. To date, most trials have been non-randomised and, often non-controlled, making it difficult to evaluate protective efficacy [42]. The proposed trial is urgently needed to inform policy makers of any additional benefit of using IRS with DDT together with current best practice (LLINs). Indeed this is considered a priority area of research by Roll Back Malaria (http://www.rollbackmalaria.org). The trial will benefit our collaborators at the NMCP in The Gambia by providing capacity building: developing management systems, resistance monitoring strategies and providing training on IRS according to the principles laid down by WHO [43]. This is particularly timely since a major IRS programme with DDT is underway. The findings from this study will provide valuable information to inform policy decisions, both in this region and elsewhere, regarding how best to use scarce health care resources to control malaria most effectively.

## Abbreviations

**CDC**: Centers for Disease Control and Prevention; **CRF**: Case report form; **CV**: coefficient of variation; **DDT**: Dichloro-diphenyl-trichloro-ethane; **EC**: Ethical committee; **EIR**: Entomological inoculation rate; **GCP**: Good clinical practice; **GLLAMM**: Generalized Linear Latent and Mixed Models; **ICH**: International conference on harmonisation of technical requirements for registration of pharmaceuticals for human use; **ID**: Identification; **IRS**: Indoor residual spraying; **ITN**: Insecticide Treated Net; **LLINS**: Long lasting impregnated nets; **LSHTM**: London School of Hygiene and Tropical Medicine; **MRC**: Medical Research Council; **NMCP**: National malaria control programme; **PCD**: Passive case detection; **PCR**: Polymerase chain reaction; **PI**: Principal Investigator; **PHC**: Primary Health Care; **RDT**: Rapid Diagnostic Test; **SAE**: Serious Adverse Event; **URR**: Upper River Region; **VHW**: Village health worker; **WHO**: World Health Organisation; **WHOPES**: Who Pesticide Evaluation Scheme

## Competing interests

The authors declare that they have no competing interests.

## Authors' contributions

SWL and MP conceived and designed the study, and drafted the manuscript. MJ and LJ contributed to the design of the entomological collections and IRS application. LJ and BK advised on the interventions and the study communities. DJ provided the statistical input to the study. KB advised on clinical aspects of the trial. DC contributed to the design and management of the study. ML advised on the design and management of the IRS. JM reviewed the protocol. All authors read and approved the final manuscript.
